# Retinoid X Receptor as a Therapeutic Target to Treat Neurological Disorders Associated with α-Synucleinopathy

**DOI:** 10.3390/cells14100685

**Published:** 2025-05-09

**Authors:** Assylbek Zhylkibayev, Christopher R. Starr, M. Iqbal Hossain, Sandeep Kumar, Shaida A. Andrabi, Maria B. Grant, Venkatram R. Atigadda, Marina S. Gorbatyuk, Oleg S. Gorbatyuk

**Affiliations:** 1 Department of Biochemistry, School of Medicine, Wake Forest University, Winston-Salem, NC 27157, USA; 2 Department of Ophthalmology, School of Medicine, University of Alabama, Birmingham, AL 35233, USA; crstarr@uab.edu (C.R.S.);; 3 Department of Pharmacology and Toxicology, School of Medicine, University of Alabama, Birmingham, AL 35294, USAsandrabi@uab.edu (S.A.A.); 4 Department of Neurology, School of Medicine, University of Alabama, Birmingham, AL 35205, USA; 5 Department of Dermatology, Heersink School of Medicine, University of Alabama, Birmingham, AL 35233, USA; venkatra@uab.edu; 6 Department of Translational Neuroscience, School of Medicine, Wake Forest University, Winston-Salem, NC 27157, USA

**Keywords:** α-synucleinopathy, Parkinson’s disease, retinoid X receptor, α-synuclein, Lewy body, dopamine, inflammation, NURR1, PPARα

## Abstract

This study investigated the therapeutic potential of the nuclear retinoid X receptor (RXR) in mitigating the progression of alpha-synucleinopathies (αSNPs), particularly in Parkinson’s disease (PD). PD-like pathology in mice was successfully induced through the co-delivery of AAV expressing human α-synuclein (αS) and αS preformed fibrils (PFFs) into the substantia nigra pars compacta (SNpc). Significant increases in Lewy body (LB)-like inclusions, loss of tyrosine hydroxylase-positive (TH+) neurons, and reductions in dopamine (DA) levels in the striatum were observed. Additionally, diminished levels of PPARα and NURR1—proteins essential for neuronal survival—along with elevated expression of IBA1 and GFAP, markers of microglial activation and astrocytic gliosis, respectively, are associated with the pathogenesis of Parkinson’s disease. AAV-mediated overexpression of human RXRα demonstrated preservation of TH+ neurons, prevention of DA decline, and attenuation of αS accumulation. Furthermore, RXR-treated PD brains showed a reduced number of GFAP+ and Iba1+ cells, decreased GFAP+ and IBA1+ immunoreactivity, and fewer and less widespread LB-like aggregates. RXR overexpression also enhanced the production of PPARα and NURR1. These findings suggest that RXRα upregulation promotes neuroprotection by mitigating αSNPs and chronic neuroinflammation, a major contributor to PD progression. This research underscores the therapeutic potential of targeting nuclear receptors, such as RXR, in neurodegenerative diseases like PD.

## 1. Introduction

Alpha-synucleinopathies (αSNPs), including Parkinson’s disease (PD) and dementia with Lewy bodies (LB), rank as the most prevalent neurodegenerative condition after Alzheimer’s disease (AD) [1]. These conditions are characterized by abnormal intraneuronal inclusions of α-synuclein (αS) [1]. While the pathology of PD is multifaceted, involving several interconnected processes including the degeneration of dopamine (DA) nigral neurons, LB formation, mitochondrial dysfunction, protein aggregation, impaired clearance, and oxidative stress, chronic neuroinflammation stands out as a hallmark of PD pathophysiology [1,2]. The glial cell activation and heightened levels of pro-inflammatory factors constitute prevalent features of the PD-afflicted brain [3]. To date, only a few treatments exist that interfere with inflammation and immune deficiency including minocycline, dexamethasone, and several non-steroid anti-inflammatory drugs. While these approaches protect DA neurons from degeneration and glial cell activation, a breakthrough therapy based on innovative molecular mechanisms and targets is an unmet need.

The modulation of nuclear receptors (NRs) has been proposed as one of the most attractive therapeutic strategies for correcting PD pathobiology [4,5,6,7,8,9,10]. The nuclear retinoid X receptor (RXR) is of particular interest for therapeutic intervention due to its binding and activation of both permissive partners, NURR1 and PPARs, whose dysfunctions have been observed in AD, multiple-system atrophy, and PD [11,12,13] and that are known to regulate the expression of inflammation-associated genes [14,15]. Despite the importance of pharmacological RXR activation against PD-causing toxins [4,16,17,18], effective RXR-based treatments for PD patients are still in development.

Cumulative evidence indicates that abnormal RXR signaling triggers neuronal stress and neuroinflammation in PD pathobiology [6,8]. Activation of RXR binding partners, either NURR1 or PPAR, is beneficial for reversing the PD pathology seen in animal and cellular models of PD [5,7,11,15,19,20,21]. Furthermore, it has been shown that the activation of RXR by treatment with the RXR ligand LG100268 protects cultured DA neurons from stress and PD-like pathology induced by toxin 6-hydroxy dopamine (6-OHDA) [6]. The activation of PPAR by the application of fenofibrate and GW501516 reduces neuroinflammation and PD neurodegeneration [15]. Activation of the NURR1/RXRα heterodimer by NURR1 agonists can stand as a monotherapy for PD to increase striatal DA levels and upregulate NURR1 target genes in PD-like brain conditions [22]. Collectively, these studies lead us to ask whether RXR/PPAR and RXR/NURR1 complexes are destabilized in the experimental PD model and whether the delivery of exogenous RXR to the PD brain activates PPARs and/or NURR1, providing an effective interventional treatment aimed at controlling inflammatory response and reversing PD pathobiology.

Although NR agonists are powerful pharmacological compounds, many of them unfortunately exhibit off-target effects [21]. Consequently, the lack of evidence for a direct role of RXR upregulation in αS-induced neurodegeneration in animal models of PD, along with the absence of disease-related αSNPs, fueled our interest in conducting the current study, which directly targets RXR expression. Using recombinant adeno-associate virus (AAV) to overexpress human RXRα (AAV-RXR) in the mouse substantia nigra pars compacta (SNpc), we examined the role of RXR in PD pathogenesis. Specifically, we investigated the impact of RXRα on the inflammatory response and the changes in the viability of nigral tyrosine hydroxylase positive (TH+) cells in response to αS-induced neurotoxicity.

## 2. Materials and Methods

### 2.1. AAV Vectors

All AAV vectors used in this study were generated using a common plasmid backbone containing the chicken β-actin promoter to drive transgene expression, packaged in an AAV2/5 capsid and purified as described previously [23]. Titers for all AAV constructs were equalized to 6 × 10^11^ vg/mL.

### 2.2. Primary Mouse Cortical Neuron Culture

All experiments involving the use of animals were approved by the Institutional Animal Care and Use Committee (IACUC) at the University of Alabama at Birmingham. Primary mouse embryonic cortical cells were isolated at embryonic day 15 (E15) as described previously [24]. Briefly, a day before isolation, 12-well plates and glass slides were treated with the attachment factor poly-l-ornithine (#P4957, Sigma-Aldrich, St. Louis, MO, USA). Mouse brains were dissected and placed in DMEM (#11-965-084, Fisher Scientific, Waltham, MA, USA) supplemented with 20% fetal bovine serum (#A4736401, Fisher Scientific, Waltham, MA, USA) and used as the dissection medium. Cortical tissue was trypsinized using TypLE (#12-605-010, Fisher Scientific, Waltham, MA, USA) for 10 min at 37 °C. Digested tissue was homogenized by pipetting in DMEM+10% fetal bovine serum and centrifuging at 1200× *g* for 5 min. The supernatant was discarded and replaced with Neurobasal medium (#A1371001, Fisher Scientific, Waltham, MA, USA) containing B27 supplements (#A3582801, Fisher Scientific, Waltham, MA, USA). Tissue pellets were filtered through a 40 µm cell strainer (#50-146-1426, Fisher Scientific, Waltham, MA, USA) and transferred to plates and slides. On the second in vitro day (DIV 2), the growth of glial cells was inhibited by adding 50 μM 5-fluoro-2-deoxyuridine (#f0503, Sigma-Aldrich, St. Louis, MO, USA) for 3 days. AAV-GFP or AAV-RXR was also added at DIV-2, while recombinant αS pre-formed fibrils (PFFs) from Stress Marq Biosciences (#SPR-322, Victoria, BC, Canada) were added starting at DIV 5 at a concentration of 5 µg/mL and αS protein from Stress Marq Biosciences (#SPR-322E, Victoria, BC, Canada) at a concentration of 5 µg/mL. Cells were grown until DIV 14 and further harvested for analysis.

### 2.3. Intracerebral Injection of PFFs and AAV Vectors

All surgical procedures were performed using aseptic techniques and isoflurane gas anesthesia, as previously described [25]. Stereotaxic coordinates for intranigral injections in C57BL6 mice (three-month-old males, 28 ± 3 g) were AP −2.9 mm, ML +1.2 mm, and DV −4.1 mm, relative to the bregma. All AAV vectors and PFFs were injected at a volume of 0.5 µL each, with a total injection volume of 1.5 µL at a rate of 0.5 µL/min. In cases of single or dual AAV vector/PFFs injections, the total volume was adjusted to 1.5 µL using PBS. For these injections, we used a pulled glass pipette with an outer diameter not exceeding 40–45 µm to avoid needle-related damage and reduce the injury-mediated inflammatory response.

### 2.4. Behavioral Test

The cylinder test was employed to measure spontaneous forelimb use [26]. A mouse was placed in a glass cylinder and the number of times it reared up and touched the cylinder wall was measured. The wall touches were subsequently scored for the left and right paws. The data were expressed as the percentage of contralateral (left) to injected side paw usage relative to the total number of touches by both paws.

### 2.5. Isolation and Processing of Tissues

Animals were deeply anesthetized by a ketamine (50 mg/kg) and xylazine (10 mg/kg) cocktail intraperitoneally. The brains were removed and divided into two parts. The caudal part containing the SNpc was fixed in ice-cold 4% paraformaldehyde in 0.1 M phosphate buffer, pH 7.4. The fixed parts of the brains were stored overnight at 4 °C and then transferred into 30% sucrose in 0.1 M PB for cryoprotection. Thirty µm thick coronal sections were cut and further processed for immunohistochemistry. The rostral piece of brain tissue was immediately used to dissect the right and left striatum. Tissue samples were frozen separately on dry ice and kept at −80° C until assayed. In this study, the mice from the 4-week time point of post-surgery were used to obtain SNpc tissue samples for Western blot analysis. Frozen brains were sectioned on a Leica CM1510S cryostat (Leica, Buffalo Grove, IL, USA) into 150 μm slices with SNpc tissue subsequently dissected out under a microscope, as previously described [24].

### 2.6. Immunohistochemistry

For the bright-field microscopy analysis, 30 μm floating sections were preincubated with 1% H_2_O_2_–10% methanol for 15 min and then with 5% normal goat serum for 1 h. Sections were incubated overnight at room temperature with anti-TH (1:2000; #MAB318, mouse; Millipore-Sigma, Burlington, MA, USA) antibody. Next, incubation with biotinylated secondary anti-mouse antibody was followed by incubation with the avidin–biotin–peroxidase complex (VECTASTAIN^®^ Elite^®^ ABC-HRP Kit, Peroxidase (PK-6100) Vector Laboratories, Newark, CA, USA). Reactions were visualized using the NovaRED Peroxidase (HRP) Substrate Kit (Vector NovaRED^®^ Substrate Kit, Peroxidase (SK-4800), Vector Laboratories, Newark, CA, USA).

For confocal microscopy, sections were incubated with primary antibodies against human αS (1:1000; 32-8100, mouse; Invitrogen; Waltham, MA, USA), pS129 (alpha-synuclein (phospho S129); 1:500; [EP1536Y] (ab51253); rabbit monoclonal; Abcam; Waltham, MA, USA) and TH (anti-tyrosine hydroxylase antibody; 1:1000; AB1542, sheep; Millipore-Sigma, Burlington, MA, USA), human RXRα (RXRA monoclonal antibody (K8508); 1:200; 43-390-0; mouse, Invitrogen), and secondary fluorescent antibodies labeled with Alexa Fluor 488, 555, and 647 (1:500 for all; PIA32773-mouse; A21202-mouse; PIA32731TR-rabbit; PIA32794-rabbit; A21448-sheep, Invitrogen). The sections were examined using an AX-R confocal microscope coupled to a fully automated Ti2-E system (Nikon Instruments Inc., Melville, NY, USA). The NIS-Elements Software package was used for post-capture image analysis. Sequential scanning was used to suppress optical crosstalk between the fluorophores in the stationary-structure colocalization assays. All manipulations of contrast and illumination on color images as well as color replacement were carried out using Adobe Photoshop CS software (Version 23.5.5).

### 2.7. Quantitation of Phospho-Synuclein Aggregates

To quantify the phospho-synuclein aggregates, 30 μm floating brain sections were processed for immunofluorescent labeling with the pS129 antibody. To count pS129-positive inclusions, an ImageJ macro (Version 1.54) was developed by modifying a previously described macro designed for automated cell counting [27]. Briefly, the parameters of the counter were adjusted to reliably count pS129-positive inclusions. There was strong agreement between the macro-counted sections and those counted manually. To count the aggregates in the macro, an area of interest was drawn on a micrograph within ImageJ, and then the macro was run to count the total number of aggregates in the outlined area. The area occupied by aggregates was manually quantified in ImageJ using the freehand tool and the measure function.

### 2.8. Unbiased Stereology

The unbiased stereological estimation of the total number of TH+ neurons in SNpc was performed using the optical fractionator method as described previously [28]. The sampling of cells to be counted was performed using the Micro Brightfield Stereo Investigator System. The software was used to delineate the transduction area at 4× on 30 µm sections and generate counting areas of 100 × 100 µm. The estimate of the total number of neurons and coefficient of error due to the estimation was calculated according to the optical fractionator formula [28].

### 2.9. DA Measurements

Frozen striatal tissue samples were shipped on dry ice to the Neurochemistry Core Lab at Vanderbilt University Medical Center, Nashville, TN for HPLC analysis of DA, 3,4-dihydroxyphenylacetic acid (DOPAC) and homovanillic acid (HVA) in the striatum. A total of 5–6 animals per group were analyzed for striatal DA metabolites.

### 2.10. Western Blot Analysis

Brain tissues were collected and lysed with RIPA buffer (Cell signaling, 9806; Danvers, MA, USA) supplemented with 1% Halt Protease and Phosphatase Inhibitor Cocktail (Thermo Fisher Scientific, 87786, Waltham, MA, USA). Samples were homogenized using a pestle (Fisherbrand™ RNase-Free Disposable Pellet Pestles; 12-141-368; Fisher, Scientific, Waltham, MA, USA), rotated at 4 °C for 30 min, and centrifuged at 4 °C with 12,000× *g* speed for 15 min. Based on the Bradford method, the supernatant was used for protein quantitation using a protein assay (Bio-Rad Protein Assay Kit I; 5000001; Hercules, CA, USA). A total of 80–100 µg of protein was loaded onto 4–20% Mini-PROTEAN^®^ TGX™ Precast Gel (Bio-Rad, 4561093EDU, Hercules, CA, USA) and transferred to polyvinylidene difluoride membrane (Biorad, 1704272, Hercules, CA, USA) using the Trans-Blot Turbo Transfer System (BioRad, 1704150, Hercules, CA, USA). Membranes were incubated for 1 h in 5% skim milk (Bio-Rad, 1706404, USA) prepared with 1X Tris buffered saline (Bio-Rad, 1706435, Hercules, CA, USA) with 0.1% Tween 20 (Millipore-Sigma-Aldrich, P1379, Burlington, MA, USA). Primary antibodies were diluted in 5% bovine serum albumin (Fisher, BP9703-100, USA) dissolved in 1X TBS. The following primary antibodies were used in the study: mouse anti-αS (1:2000; BD Transduction Laboratories, #610787, Milpitas, CA, USA), mouse anti-TH (1:2000; #MAB318, Millipore-Sigma, Burlington, MA, USA), and anti-hRXRα mouse (1:200; Abcam #ab50546, Waltham, MA, USA). Secondary antibodies (1:10,000 for all) were purchased from LI-COR Biosciences, Lincoln, NE, USA: HRP goat anti-rabbit IgG (926-80011); HRP goat anti-mouse IgG (926-80010). Images of membranes were captured and analyzed using the Odyssey XF system (LI-COR, Biosciences, Lincoln, NE, USA).

### 2.11. Statistical Analysis

Data were analyzed using a Student *t* test as well as one- and two-way analysis of variance with Tukey’s multiple comparisons tests using Prism 10 (GraphPad Software, Inc., Boston, MA, USA). Data were presented as the mean ± SEM. “N” represents the number of animals used in the experiment. Each experimental group consisted of 4 to 14 mice.

## 3. Results

### 3.1. Development of PD-like Mouse Model Associated with αS-Induced Neurodegeneration

**Figure 1:**
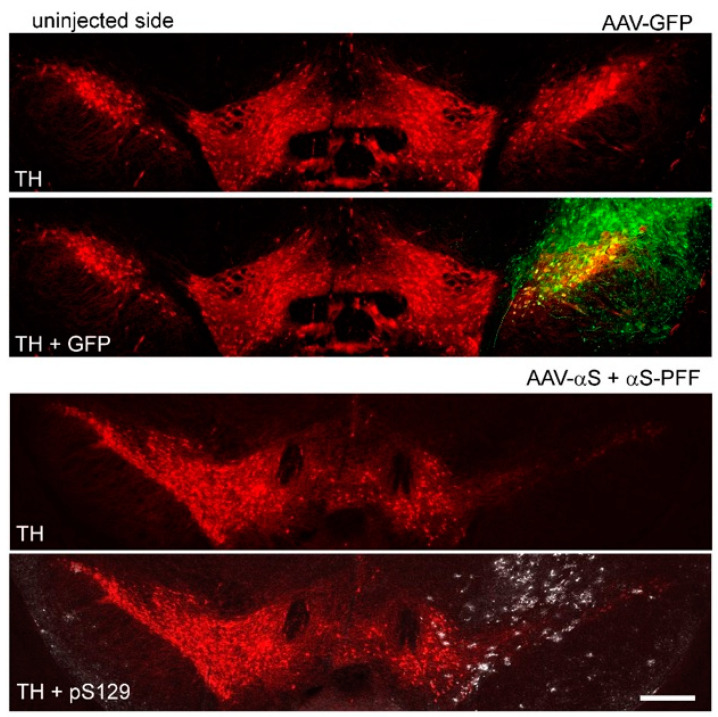
Fluorescent microscopy of the mouse SNpc at 8 weeks after the AAV-GFP and AAV/PFF injections. The upper image shows the expression of the GFP transgene on the injected side of the mouse brain from the control group. GFP (green) was expressed in most TH+ neurons (red) in SNpc and can be seen in the SN pars reticulata as well as surrounding neuronal structures of the mesencephalon. The bottom image illustrates pS129-positive inclusions (white) in the SNpc neurons of mice from the experimental group with the combined injection of AAV/PFF. Scale bar: 0.5 mm.

In this study, we employed an animal model of PD-like pathology, as previously described by Thakur et al. and Bjorklund et al. [29,30]. In order to mimic the PD pathogenesis in C57BL6 adult male mice, we used combined injections of AAV-αS and PFFs (AAV/PFF) into the SNpc. The PFFs were purchased from Stressmarq (SPR-322) and the total amount of injected PFFs was 2.5 µg (in 0.5 µL). The AAV containing a blank expression cassette, which is an αS vector with an early stop codon, served as a control virus (BV) [24,31]. Monomeric αS (Stressmarq; SPR-321) was used as a control substance to the PFFs. A mixture of BV and monomeric αS (BV/αS) served as a control for the combined AAV/PFF administration. Mice were injected with AAVs and/or PFFs on one side of the brain; the other side was kept as a non-transduced internal control (Figure 1).

**Figure 2:**
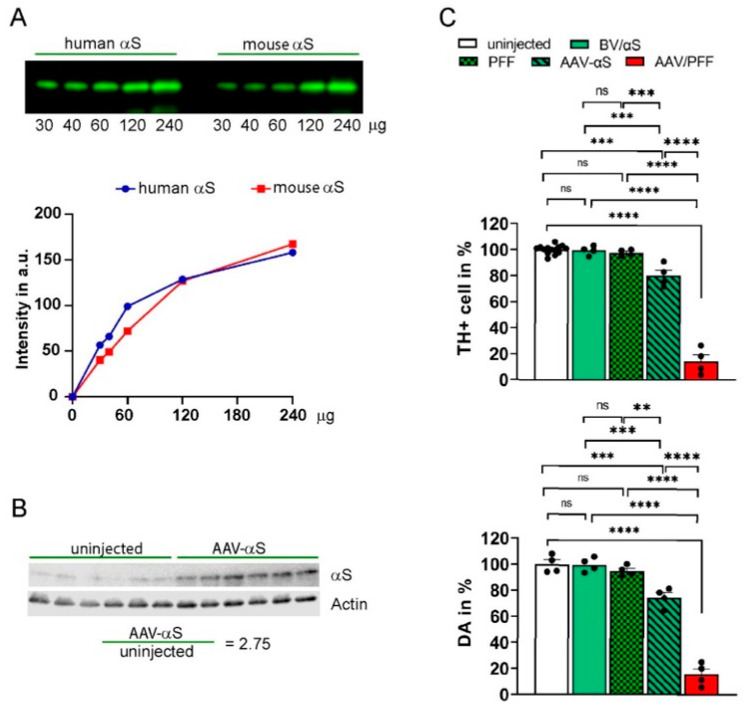
Comparative analysis of αS mouse models of PD. All AAV vectors had the same volume and titer (6 × 10^12^ vg/mL). The total injection volume (2 μL) of AAV-GFP and AAV-αS was adjusted using PBS to equalize with BV/αS or AAV/PFF. (**A**) The comparative affinity test of the anti-αS antibody to bind recombinant human and mouse αS proteins of known concentration used in the study demonstrated similar affinity of anti-αS antibody to bind both recombinant proteins. (**B**) Measurement of the total αS expression in animals injected with AAV-αS alone at 4 weeks post-injection. Western blot (WB) using the anti-αS antibody tested in (**A**) demonstrated the comparative expression of αS in the nigral tissues of the uninfected and injected sides. The amount of αS was normalized to β-actin, and the ratio of the injected versus uninjected sides was calculated (*n* = 6). (**C**) Combined AAV/PFF administration into SNpc resulted in more severe loss of nigral TH+ cells and striatal DA compared with individual injections of AAV-αS or PFF at 8 weeks post injection (images presented in the Figure S1). The upper graph demonstrates unbiased estimation of nigral TH+ cells in the SNpc of mice injected with AAV-αS or PFF and a combination of AAV/PFF or BV/αS. Tissue slices were labeled with antibody to TH as described in the Materials and Methods, and the percentage of surviving cells was calculated by comparison with the uninjected side in the same animal. Lower graph shows the measurement of striatal DA on the injected and uninjected sides of individual animals expressed as the mean percentage of DA remaining on the injected side. Both graphs demonstrated the significant loss of nigral TH+ cells and striatal DA in AAV/PFF injected mice at 8 weeks post-injection. One-way group ANOVA statistics with Tukey’s multiple comparison test is indicated as **, ***, and **** = *p* < 0.05, *p* < 0.01, *p* < 0.001, and *p* < 0.0001, ns—no significant, *n* = 4 for each group except of TH+ cell count in the uninjected control, which was *n* = 14. Abbreviations: αS, alpha-synuclein; DA, dopamine; AAV, recombinant adeno-associate virus; BV, control blank expression virus; SNpc, substantia nigra pars compacta; TH, tyrosine hydroxylase; AAV/PFF, combined AAV-αS and PFFs injection; BV/αS, combined BV and monomeric αS injection, served as the control to AAV/PFF.

The study involved several groups of animals: the uninjected controls and those injected with single AAV-αS, BV, AAV-GFP, AAV-RXR, and a combination with AAV/PFF or BV/αS. We established injection conditions to secure the αS expression level, and to maintain consistency with the reported αS levels in postmortem human PD brains [32], we used the AAV-αS titer that induced moderate levels of αS overexpression, which was threefold higher relative to the endogenous levels. To confirm this, we first validated a mouse anti-αS antibody capable of recognizing an epitope shared by both endogenous mouse αS and exogenous human αS (Figure 2A). In these experiments, the ratio of αS in the injected versus uninjected mouse SNpc was measured at 4 weeks post-injection, a time point when neurodegeneration was not yet significant. The αS ratio varied between 2.6 and 3.1, with an average increase of 2.75-fold in total αS expression (Figure 2B).

We observed significant striatal dopamine (DA) and TH+ neuronal loss at 8 weeks post-injection. These effects were most dramatic in mice injected with AAV/PFF compared with those receiving either rAAV-αS or PFFs alone (Figure 2C), highlighting the advantages of the combined approach in accelerating the development of LB-like features. Notably, no differences were observed between the uninjected mice and BV/αS injected controls nor between the control groups and PFF injections alone. However, substantial differences were detected between the control groups and AAV-αS as well as between the control groups or AAV-αS and AAV/PFF (Figure 2C). As a result of the AAV/PFF injections, we observed the formation of pS129-positive inclusions (Figure 1). These inclusions were associated with increased neuronal vulnerability, which was seen as a dramatic reduction in the number of TH+ cells in the SNpc accompanied by a decline in striatal DA levels (Figure 2C).

### 3.2. Combined AAV/PFF Injection Induce Severe Neurodegeneration Accompanied by Inflammatory Response and Gliosis

To investigate a link between the observed aggregates, TH+ cell loss, and inflammation, we next analyzed the level of glial fibrillary acidic protein (GFAP). GFAP is upregulated in response to CNS injury or disease and serves as a marker of astrogliosis. Elevated GFAP levels are observed in conditions such as AD, PD, HD, and ALS and contribute to neurodegeneration via pro-inflammatory signaling [33,34,35]. During activation, accompanied by upregulated GFAP expression, astrocytes release pro-inflammatory cytokines and chemokines (e.g., IL-1β, TNF-α) that promote immune cell recruitment [34]. Another protein, IBA1, serves as a marker for microglia and their activation, reflecting microglial dynamics in CNS health and disease [35]. Its upregulation in activated microglia makes it a critical tool to study neuroinflammation. Activated microglia release cytokines such as IL-1β, TNF-α, and IL-6, which can exacerbate neuroinflammation. Therefore, we analyzed these markers in the PD brains.

**Figure 3:**
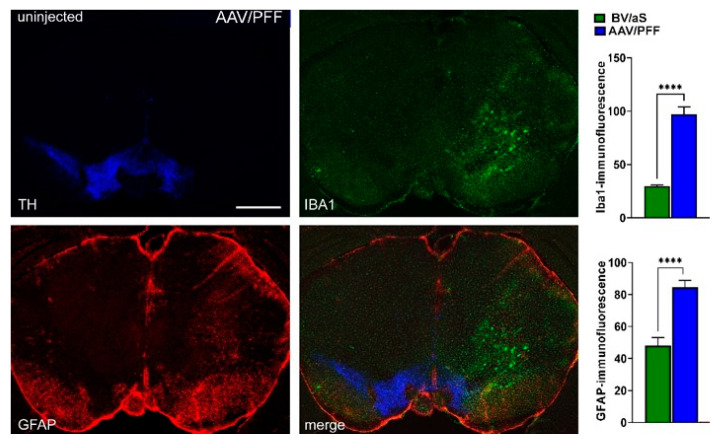
At 8 weeks post AAV/PFF injection, a significant increase in GFAP and IBA1 immunofluorescence was observed in the mouse brain, indicating robust astrocytic and microglial activation, respectively. Student *t* test was used for comparison: **** *p* < 0.0001 (*n* = 5–6). Scale bar: 1 mm.

Figure 3 demonstrates a significant increase in both the GFAP and IBA1 fluorescent signals ranging from 2- to 3-fold in the AAV/PFF injected brains previously identified with dramatic TH+ cell loss compared with BV/αS injections. Results of this experiment indicated a notable inflammatory response associated with the combined AAV/PFF approach.

### 3.3. RXR Diminishes LB-like Formation in Primary Mouse Cortical Neurons

**Figure 4:**
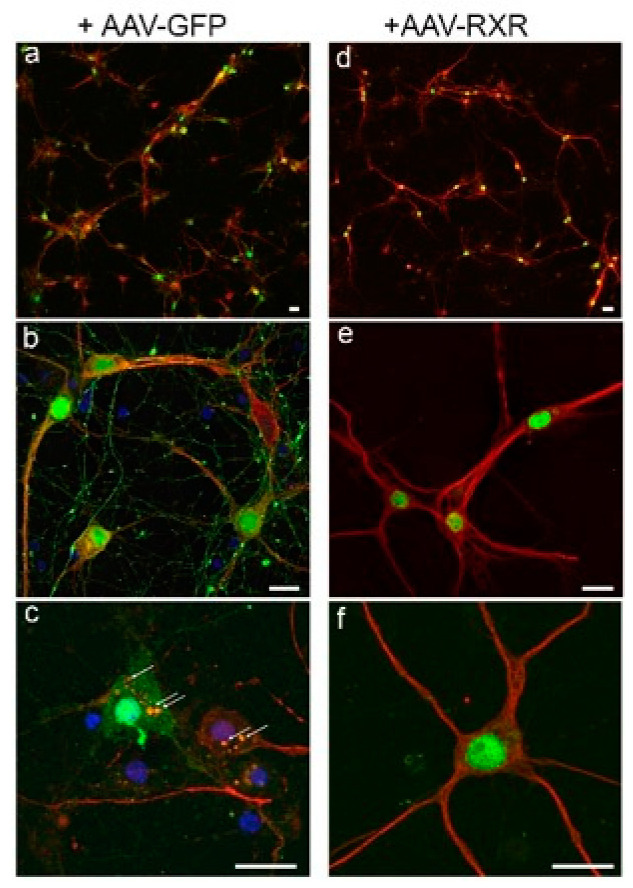
Confocal images illustrating the AAV-mediated expression of native GFP (**a**–**c**) and human RXRα (**d**–**f**), both shown in green, in the cortical neurons at DIV 14 after nine days of combined exposure to PFFs and monomeric αS. Both experimental groups demonstrated immunofluorescent staining with the anti-pS129 antibody (shown in red). However, LB-like pS129-positive inclusions localized to the cell bodies denoted by arrows were identified only in the GFP-positive cells and none of them were found in the hRXRα expressing neurons. DAPI staining (in blue) added in (**c**). Scale bars: 25 μm.

Preliminary testing of the AAV-RXR vector in the primary mouse neuronal cells (Figure 4) demonstrated a powerful RXR effect on the formation of 129-positive LB-like inclusions. Despite diffuse pS129 immunostaining observed in both the GFP and RXR experimental groups, LB-like αS inclusions (denoted by arrows) localized to the cell bodies were identified only in the GFP-positive cells after nine days of exposure to PFFs and monomeric αS. None of these inclusions were found in the hRXRα-expressing neurons, suggesting the therapeutic potential of treatment with exogenous RXR.

### 3.4. RXR Overexpression Significantly Ameliorates PD-Associated Pathology in Mouse PD Model

**Figure 5:**
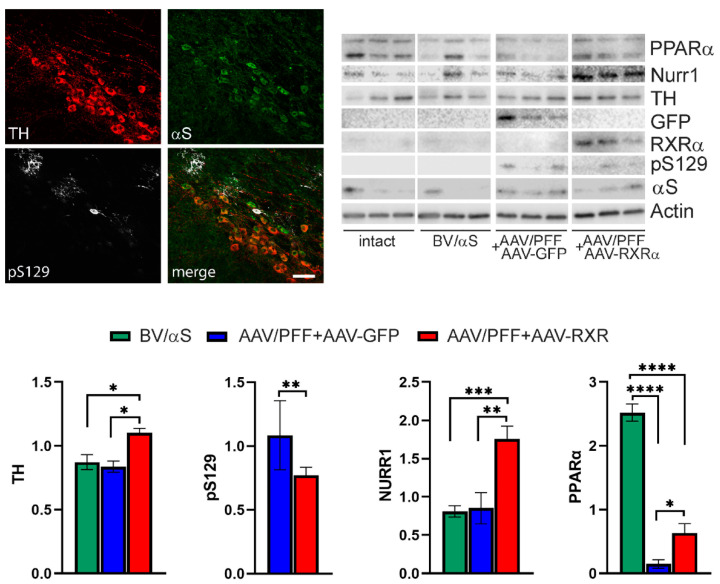
The SNpc of mice at 4-weeks after combined AAV/PFF injection. Confocal imaging (**upper left**) revealed pS129-positive aggregates (shown in white) in the TH+ neurons (shown in red) of the SNpc in mice treated with AAV/PFF. Exogenous human αS is shown in green. Images of the WB membranes probed with different antibodies are shown in the **upper right**. The calculation of protein expression is shown in graphs. The amount of analyzed proteins was normalized to β-actin (except of pS129) and shown in graphs in arbitrary units. WB analyses revealed no difference in the nigral TH protein levels between the control group (BV/αS) and AAV/PFF + AAV-GFP injected brains at 4 weeks. However, the TH protein level was increased upon treatment with AAV-RXR (AAV/PFF + AAV-RXR) in the nigras. The levels of PPAR were significantly downregulated in the AAV/PFF + AAV-GFP injected brains compared with BV/αS, although the same samples showed no difference in NURR1. Alternatively, in the AAV/PFF + AAV-RXR treated nigras, a significant increase in both PPAR and NURR1 was observed upon the treatment with AAV-RXR (*n* = 6). The level of pS129 protein adjusted to total αS was also significantly upregulated in AAV/PFF + AAV-RXR compared with the AAV/PFF + AAV-GFP mice at 4 weeks. Student *t* test is indicated as ** = *p* < 0.01, *n* = 5. One-way ANOVA was used with Tukey’s multiple comparisons test and presented as ±SEM (* *p* < 0.05, ** *p* < 0.01, *** *p* < 0.001, **** *p* < 0.0001; Scale bar: 25 μm.

**Figure 6:**
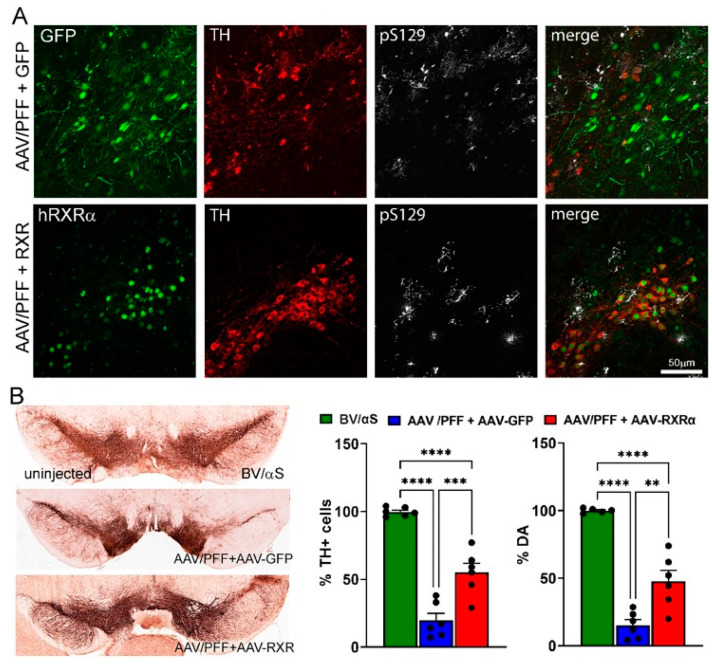
The SNpc of mice at 8-weeks after combined AAV/PFF injection. AAV-mediated overexpression of human (h) RXRα ameliorates PD-like pathology induced by AAV/PFF. (**A**) Confocal images demonstrate the expressions of transgenic GFP or hRXRα (in green) within the TH+ neurons (in red) with pS129 deposits (in white). (**B**) Bright-field images showing the TH+ cells in the SNpc of representative animals from the control and experimental groups. These sections were labeled with standard DAB immunohistochemistry (see Materials and Methods). Graphs demonstrate the number of nigral TH+ cells remaining on the injected side compared with the uninjected side ±SEM. The striatal DA levels shown as a percentage of injected vs. uninjected sides. One-way group ANOVA statistics with Tukey’s multiple comparison test is indicated as **, ***, and **** = *p* < 0.01, *p* < 0.001, and *p* < 0.0001; *n* = 6 (all groups).

Previous studies have indicated that reduced RXR signaling triggers neuronal stress and neuroinflammation in PD pathobiology [6,8]. To explore the therapeutic potential of RXR activation for PD treatment, we utilized AAV-mediated transduction to deliver either human RXRα or the control GFP, alongside AAV/PFF, into the mouse SNpc. Various control and experimental groups were included in the study, incorporating combinations of GFP, human RXRα viral vectors, and control BV/αS. The mice were analyzed at 4- and 8-weeks post-injection (Figure 5 and Figure 6). An unbiased stereology count of the nigral TH+ cells in the injected SNpc was compared with the uninjected SNpc for each mouse. No significant differences were observed between animals injected with the GFP, human RXRα vectors, and/or control BV/αS, and the uninjected side at both the 4- and 8-week time points (*p* = 0.963). Therefore, the BV/αS control group at both time points was used for further comparisons.

Figure 5 presents the study results of the mouse SNpc terminated at 4 weeks post-injection with AAV/PFF. Despite confocal imaging revealing pS129-positive inclusions in the TH+ neurons in the SNpc and surrounding nervous structures of mice treated with AAV/PFF, no significant nigral TH+ cell loss or marked reduction in striatal DA was detected in the mouse brains at this point. However, a notable reduction in the PPARα levels was observed in the nigral tissue of the AAV/PFF-injected mice at 4 weeks post-injection. At the same time, the co-injection of AAV/PFF with AAV-RXR led to an over 2-fold increase in the PPARα level compared with the AAV/PFF + AAV-GFP injected mice. In addition to PPARα, the RXR overexpression in the AAV/PFF-injected mice enhanced NURR1 production in the SNpc at 4 weeks post-injection. This implies that RXRα overexpression in the PD-like brain activates NURR1, which has been proposed as a standalone therapeutic target for PD [22] Consistent with a previous study demonstrating NURR1-induced TH production [17], this NURR1 upregulation was accompanied by a significant increase in TH protein. Moreover, the level of pS129 protein was significantly downregulated (*p* < 0.001) in AAV/PFF + AAV-RXR compared with the AAV-PFF + AAV-GFP mice at 4 weeks post-injection. This finding indicates that NURR1 is a downstream target of RXR activation and underscores the potential of RXR-mediated NURR1 therapy.

**Figure 7:**
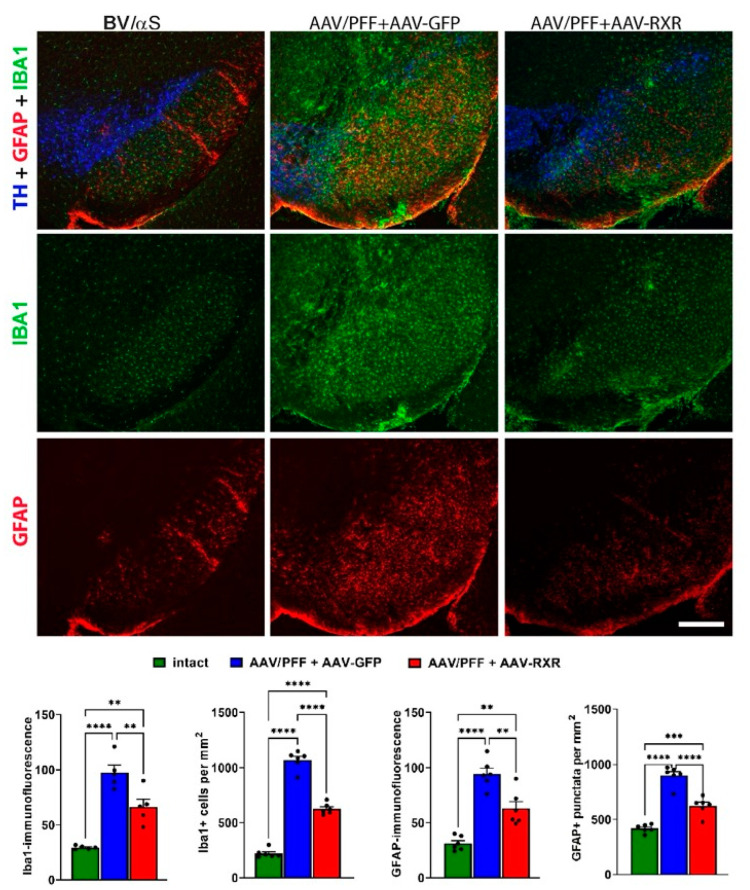
RXRα overexpression ameliorates inflammation response in the SN of mice at 8-weeks after combined AAV/PFF injection. Confocal images (**upper**) illustrate the reduced expression of inflammatory markers, GFAP (in red) and IBA1 (in green), in AAV/PFF brains upon AAV-RXR delivery. TH+ cells are shown in blue. The graphs (**bottom**) depict the results of the immunofluorescent detection of GFAP and IBA1 expression at 8 weeks. RXR-treated PD brains demonstrated a reduced number of GFAP+ and IBA1+ cells and decreased GFAP+ and IBA1+ immunoreactivity. One-way ANOVA analysis with Tukey’s multiple comparisons test is indicated as **, ***, **** = *p* < 0.05, 0.01, 0.001, 0.0001; *n* = 6. Scale bar: 0.5 mm.

At 8 weeks post-injection, while the AAV/PFF treatment induced a significant loss of nigral TH+ cells and striatal DA, the simultaneous delivery of human RXRα and AAV/PFF diminished TH+ cell loss and increased the DA level (Figure 6). Additionally, RXRα overexpression led to a decrease in GFAP immunoreactivity and GFAP+ punctata, indicating a reduced recruitment of astrocytes to the AAV/PFF-affected area at 8 weeks post-injection (Figure 7). It is also noteworthy to mention that the microglial marker IBA1 was diminished in RXR-overexpressing brains with pS129-positive inclusions, further supporting the anti-inflammatory effects of RXRα overexpression.

Motor behavior impairment was assessed using the cylinder test to measure asymmetry in forelimb use. At 4 weeks post-injection, no significant differences were observed between the control and experimental groups (Figure S1). However, by 8 weeks post-injection, the AAV/PFF + AAV-GFP group exhibited significant motor impairment compared with the control BV/αS group. In contrast, mice injected with AAV/PFF + AAV-RXR showed a trend toward improvement relative to the AAV/PFF + AAV-GFP group, though this difference did not reach statistical significance (Figure S2).

**Figure 8:**
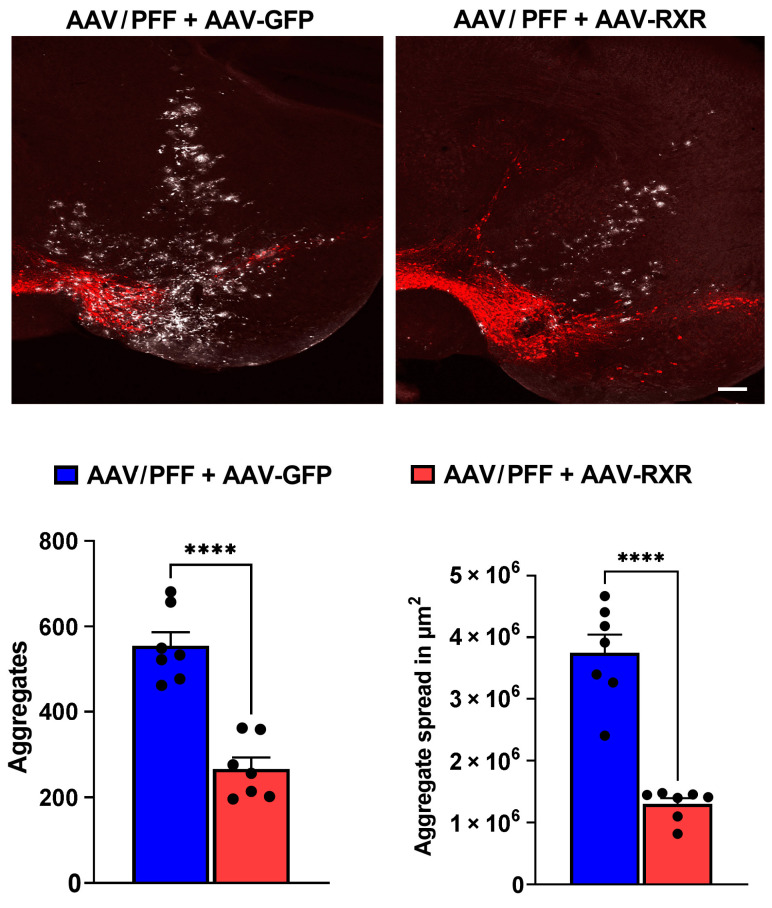
The pS129-positive inclusions (white) at the injection sites of mouse brains at 8 weeks after AAV/PFF + AAV-GFP and AAV/PFF + AAV-RXR delivery. TH-positive cells are stained in red (**upper panel**). Graphs represent the comparative count and distribution area of the pS129-positive aggregates (**bottom panel**). Unpaired *t* test is indicated as **** = *p* < 0.01, *n* = 7.

Finally, we analyzed the distribution area of pS129-positive inclusions affected by RXR overexpression by comparing the injection sites of the AAV/PFF + AAV-GFP and AAV/PFF + AAV-RXR mice. We counted the pS129-positive inclusions in outlined areas (Figure 8). Remarkably, the number of aggregates and the size of the area containing pS129+-LB-like aggregates, measured in μm^2^, significantly decreased with the distance from the point of injection in the AAV/PFF + AAV-RXR injected brains compared with the AAV/PFF + AAV-GFP injections (*p* < 0.0001).

## 4. Discussion

In the current study, we presented a reliable mouse model that mimics PD-like pathology including the accumulation of LB-like aggregates, TH neuronal loss, DA deficit, and activation of astrocytes and microglia. Our data indicate that a combination approach delivering AAV-αS and commercial PFFs to the SNpc resulted in the activation of a severe inflammatory response. This approach significantly accelerated and mimicked PD progression compared with previously reported models based on individual AAV-αS or PFF injections. This confirms the observation by Bjorklund A et al. [30] that the combined AAV/PFF delivery offers several advantages over the standard PFF model due to the enhanced and accelerated αS pathology and glial response induced by the PFFs and elevated αS levels, as shown in Figure 2C and Figure 3. An advantage of the current study is that we demonstrated that the AAV/PFF-mediated neurodegeneration provides a window of opportunity for drug delivery according to our data. A limitation of the current study is that the RXR-based intervention in the PD pathogenesis should be tested at later stages with marked disease progression, thus mimicking more of the real-life situation in individuals with PD.

The AAV/PFF mouse model provides benefits by triggering an early inflammatory response observed at 3–4 weeks post-injection before severe aggregate formation is observed, as previously reported [30]. Notably, the significant striatal DA and nigral TH+ cell loss was not yet detected at this time point; however, a level of protein expression induced by AAV-mediated gene transfer reached a maximum [25,36,37]. This point allowed us to the study pathogenic mechanisms triggered by AAV/PFF.

Our data indicate that the overexpression of human RXRα in the SNpc ameliorates αS-associated neurodegeneration. This was evident through a diminished loss of striatal DA and nigral TH+ cells, and the reduced deposition of pS129-positive aggregates. Furthermore, we revealed a reduced GFAP and IBA1-positive fluorescent signaling and number of positive cells. Given the limitation of the current study, including the lack of a detailed analysis of activated astrocytes and microglia phenotypes, this reduction suggests fewer recruited astrocytes and IBA1-positive microglial cells, overall indicating a mitigation of neuroinflammation. In agreement with diminished neuroinflammation, the reduction in αS deposits in the PD brain also suggests that RXR upregulation prohibits the aggregation of an insoluble form of the protein. The results of TH+ cell counting and striatal DA assay aligned, indicating the neuroprotective effects of RXR upregulation.

Based on our data, we anticipate that PPAR is necessary for RXR-based therapies to enhance TH cell survival and DA levels in the PD brain. We revealed that AAV/PFF treatment downregulates PPARα production. In turn, RXR overexpression induces a significant upregulation of PPARα. It is well-known that PPAR α, β, and γ are the permissive RXR binding partners; when activated in the central nervous system, RXR plays a critical role [9]. Recent studies have revealed a reduced PPARα expression in AD brains [38] and the genetic linkage to the disease [39,40]. The neuroprotective effect of PPAR agonists have recently been proposed using neurological murine models and include anti-inflammatory effects, APP degradation, and Aβ inhibitory functions [41,42,43,44,45] The pharmacological modulation of PPARs by pioglitazone, rosiglitazone, ibuprofen, piroxicam, ciglitazone, and GW1929 has been shown to be neuroprotective in AD, ALS, MS, and PD models [46,47,48,49,50,51]. In particular, in MPTP and 6-OHDA models of PD, preclinical studies have shown that different PPARγ agonists reduce neuronal cell death, microglial activation, and the DA level in the hippocampus and SNpc [15,19,52,53,54]. A conformational change in the RXRa/PPARγ complex the heterodimer to bind PPAR-response elements (PPREs) in the nucleus to activate gene transcription. They can both directly upregulate genes involved in lipid metabolism and downregulate genes involved in inflammation [15,51,53]. Therefore, it is not surprising that our results demonstrated that the AAV-mediated overexpression of human RXRα increases the expression of PPARα in mouse brains with PD-like pathology.

RXRα and PPARα, when activated, influence the transcription of genes related to inflammation and oxidative stress by acting as transcription factors that bind to specific DNA sequences called PPREs (peroxisome proliferator response elements). They can both directly upregulate genes involved in lipid metabolism and downregulate genes involved in inflammation.

Our data also indicate that in addition to PPARα, RXR overexpression in mice with experimental α-synucleinopathy enhances NURR1 production in SNpc. This finding is in accordance with multiple studies that have highlighted the significance of changes in NURR1 expression during the pathogenesis of PD and related disorders. Thus, it has been shown that NURR1 is significantly decreased in the PD nigral neurons containing αS-immunoreactive inclusions and in the DA nigral neurons with neurofibrillary tangles, correlating with the loss of TH and diminished intracellular pathology in both αSNP and tauopathies [55]. Furthermore, the downregulation of NURR1 promoted the upregulation of the NF-κB/NLRP3 inflammasome axis [56,57,58], while NURR1 overexpression and activation by means of the HX600 agonist treatment had significant anti-inflammatory effects, decreasing the expression of genes involved in inflammation control [59]. Nurr1 mainly acts as an anti-inflammatory agent, especially in the microglia and astrocytes, by repressing pro-inflammatory cytokines. It does this by binding to NF-κB-p65 on inflammatory gene promoters and recruiting the CoREST corepressor, which silences gene expression [17]. It is also worth mentioning that the TH upregulation and anti-inflammatory therapeutic effect from the application of the HX600 agonist is associated with the activation of the NURR1/RXR complex, suggesting their dual role in PD development.

In addition, a most recent study has shown that activated RXR can affect αS proteostasis in PD patient-derived neurons, stimulating the lysosomal clearance of αS [60].

In the current study, the PD mouse model limited us to using a loss-of-function approach. The model exhibited extensive neurodegeneration, with an 80–85% loss of TH⁺ neurons and a 90–95% reduction in the striatal DA levels. Under these conditions, any additional detrimental effect from RXR inhibition would likely be biologically negligible. A marginal difference between 85 and 95% and complete (100%) degeneration is unlikely to be statistically significant or mechanistically informative.

In summary, the current study not only validated a PD-like mouse model using commercially available PFFs, making it convenient to study the role of genetic modifiers, but also provides evidence that mice with progressive αSNPs present a model for druggable intervention in PD progression. Our study demonstrated the power of RXRα-based therapy to mitigate PD progression by targeting major pathological events such as the formation of LB-like structures, loss of TH+ neurons, and neuroinflammatory response. The mechanism of RXR-based intervention requires future investigation. At this point, it is challenging to anticipate whether enhanced RXR will demonstrate a preference for binding to one nuclear receptor over another. Moreover, the RXR-based therapy may enhance the pool of RXR homodimers available to partner with both NURR1 and PPARs to regulate the expression of genes responsible for anti-inflammatory action.

## Data Availability

The research data are available upon request. The raw data are also available as Supplemental Materials.

## Supplementary Materials

The following supporting information can be downloaded at: https://www.mdpi.com/article/10.3390/cells14100685/s1, Figure S1: Brightfield and confocal images of the SNpc in 8 weeks postinjection with PFFs, AAV-αS and AAV/PFF. Images illustrate graphs in Figure 2C. Fluorescent figures show pS129-positive inclusions (in white), TH cells (in red). Scale bar: 100 μm; Figure S2: Cylinder test. Two-way ANOVA was used. Tukey’s multiple comparisons test is presented as ±SEM (*** *p* < 0.001; *n* = 6).
