# Supplementary material for: Retinoid X Receptor as a Therapeutic Target to Treat Neurological Disorders Associated with α-Synucleinopathy
Source: Cells. 2025 May 9;14(10):685. doi: 10.3390/cells14100685 (PMC12109830; doi:10.3390/cells14100685)
Supplement: Supplementary file 1 [file cells-14-00685-s001.zip › cells-3554139-supplementary.pdf]

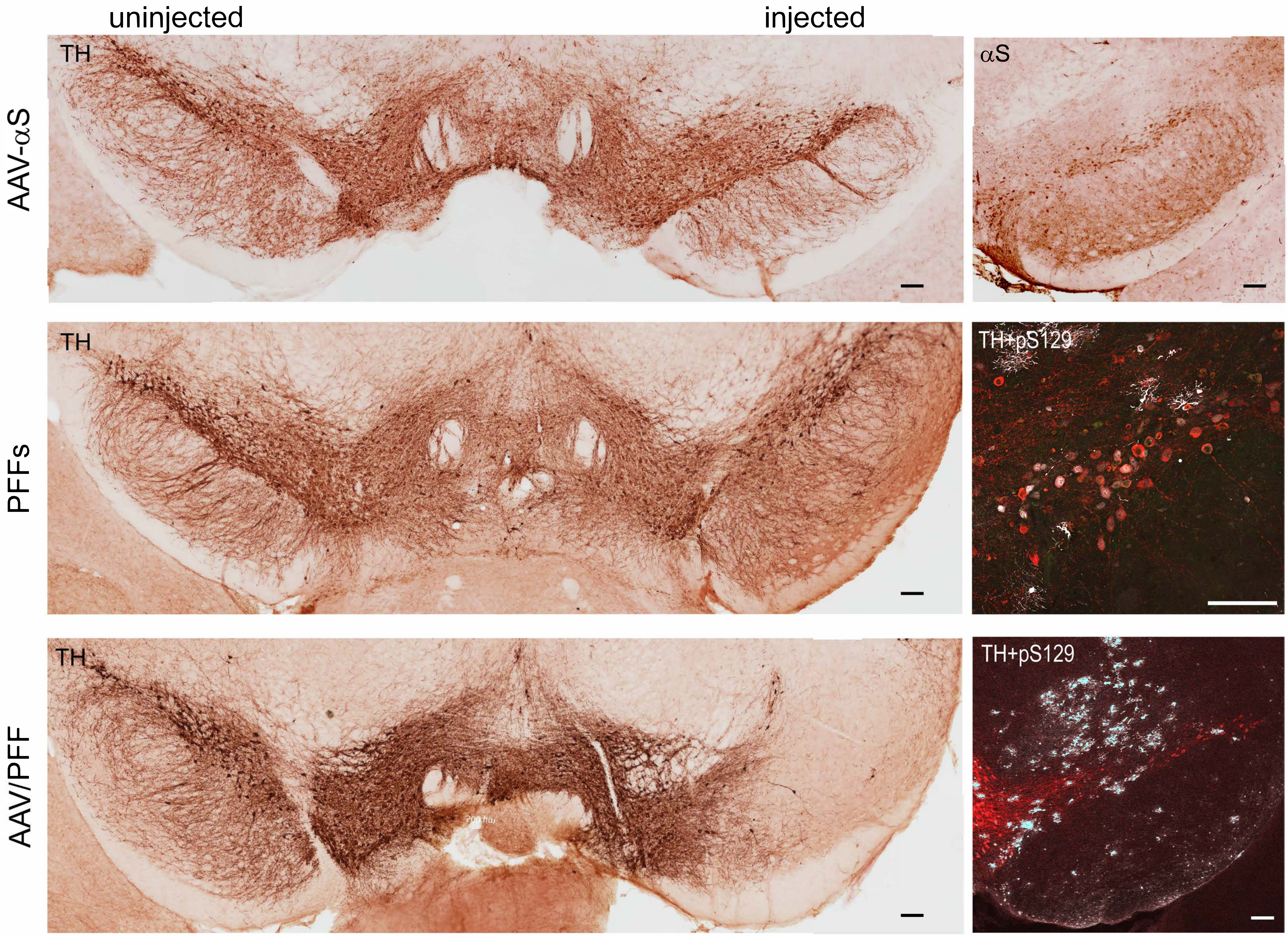

Figure S1. Brightfield and confocal images of the SNpc in 8 weeks postinjection with PFFs, AAV-αS and AAV/PFF. Images illustrate graphs in Figure 2C. Fluorescent figures show pS129-positive inclusions (in white), TH cells (in red). Scale bar: 100μm.

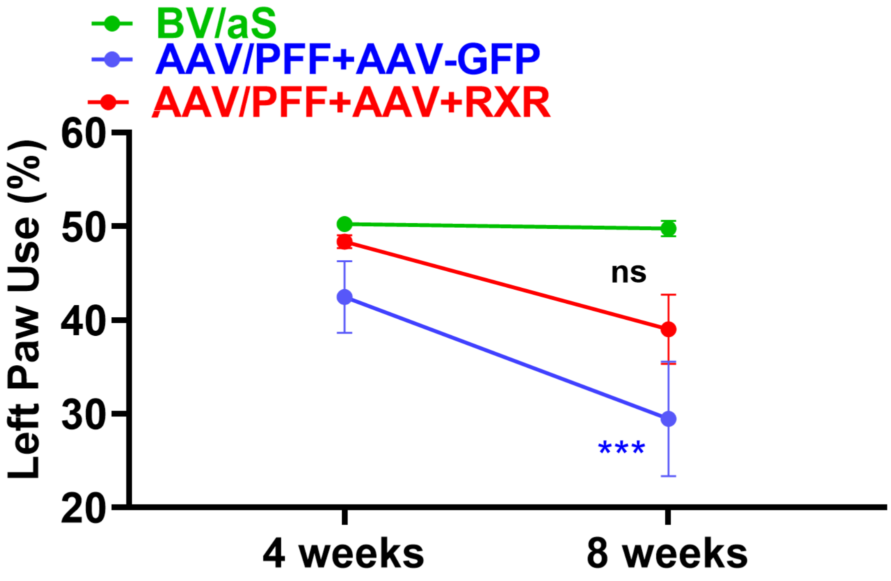

Figure S2. Cylinder test. Two-way ANOVA was used. Tukey's multiple comparisons test is presented as ±SEM (***$p < 0.001$; $n = 6$).
